# Neurodevelopmental outcome in children with congenital cytomegalovirus infection: a systematic review and meta-analysis protocol

**DOI:** 10.1186/s13643-026-03288-6

**Published:** 2026-08-08

**Authors:** Serena Salomè, Carmine Vincenzo Lambiase, Giacomo Cavallaro, Eduardo Villamor, Francesco Raimondi

**Affiliations:** 1 Division of Neonatology, Department of Translational Medical Sciences, University of Naples Federico II, Naples, Italy; 2 Department of Humanities, University of Naples Federico II, Via Porta di Massa 1, Naples, 80133 Italy; 3 Neonatal Intensive Care Unit, Fondazione IRCCS Ca’ Granda Ospedale Maggiore Policlinico, Milan, Italy; 4 Maastricht University Medical Centre, Maastricht, Netherlands

**Keywords:** Congenital cytomegalovirus infection, CCMV, Neurodevelopmental assessment, Neurodevelopmental outcomes, Developmental delay

## Abstract

**Background:**

Congenital cytomegalovirus (cCMV) infection is the most common cause of congenital infection worldwide. Neurodevelopmental sequelae have been reported in children with cCMV across different developmental stages, from infancy to adolescence. However, the evidence on the involved neurodevelopmental domains and the severity of adverse outcome remains unclear and not well defined. This protocol describes a systematic review and meta-analysis aimed at evaluating neurodevelopmental outcome in children with cCMV aged 18 years old or below and identifying predictors associated with adverse neurodevelopmental trajectories.

**Methods:**

This systematic review and meta-analysis protocol will be conducted in accordance with the Preferred Reporting Items for Systematic Review and Meta-Analysis Protocols (PRISMA-P) guidelines. The final systematic review and meta-analysis will be conducted and reported according to the PRISMA statement. Electronic searches will be performed in Embase (via Ovid), MEDLINE, PsycINFO, and Scopus without publication date restrictions. Studies evaluating neurodevelopmental outcomes in children with cCMV infection, classified as symptomatic or asymptomatic at birth, assessed through standardized neurodevelopmental tests, will be included. Studies investigating predictors of neurodevelopmental impairment and additional clinical characteristics associated with cCMV infection will also be considered. Study selection and data extraction will be performed independently by two reviewers. Any disagreements will be resolved through discussion and consensus; if consensus cannot be reached, a third reviewer will be consulted for arbitration. Quantitative synthesis will include pooled effect sizes, heterogeneity assessment, subgroup analyses, and publication bias evaluation.

**Discussion:**

This research will provide a comprehensive overview of neurodevelopmental outcomes in children with symptomatic and asymptomatic cCMV infection. The findings may support the implementation of structured neurodevelopmental follow-up programmes and early targeted interventions aimed at optimizing children’s neurodevelopmental outcomes.

**Systematic review registration:**

PROSPERO registration number CRD420251065006. Date of registration 03 June 2025.

**Supplementary Information:**

The online version contains supplementary material available at https://doi.org/10.1186/s13643-026-03288-6.

## Background

Congenital cytomegalovirus infection (cCMV) is the most frequent congenital infection worldwide. It can be completely asymptomatic at birth in most of the infected newborns although sequelae have been described also in this group later in life [1]. Neurodevelopmental outcome is not well defined because few studies are available and with inhomogeneous definitions of onset and classification and quantification of this outcome [2]. Furthermore, a quantitative analysis of cCMV-related predictors of neurodevelopmental outcomes is lacking.

The primary objective of this systematic review and meta-analysis is to assess the impact of cCMV on the neurodevelopmental outcomes of children ≤18 years old. The secondary objective is to understand the predictors of neurodevelopmental outcome in order to better define prognosis and address needs for anticipatory guidance and early intervention. It will be conducted if sufficient homogeneous data are available.

## Methods and analysis

This systematic review and meta-analysis protocol was developed in accordance with the Preferred Reporting Items for Systematic Review and Meta-Analysis Protocols (PRISMA-P) guidelines [3]. The protocol has been registered in the PROSPERO database (registration number: CRD420251065006). The PRISMA-P 2015 Checklist has been provided as supporting information (Additional file 1). The final systematic review and meta-analysis will be conducted and reported following the PRISMA statement.

### Eligibility criteria

The PECO elements [4] form the basis of clinical questions for this review. Studies will be selected according to the criteria outlined below.

Children aged ≤18 years old (P) diagnosed prospectively or retrospectively with cCMV infection (E) will be compared either with non-infected children or, in subgroup analyses, between symptomatic and asymptomatic cCMV populations (C), with respect to neurodevelopmental outcomes (O). Published original research articles written in English will be considered. Although this language restriction may represent a limitation, the majority of studies addressing this topic are expected to be indexed in international databases and published in English-language journals. No publication date restrictions will be applied.

#### Participants

Inclusion criteria: all the children with cCMV infection aged ≤18 years old at the time of neurodevelopmental assessment.

#### Exposure

cCMV may be diagnosed prospectively or retrospectively, using polymerase chain reaction, or nucleic acid amplification test, or viral culture of amniotic fluid via amniocentesis in utero, dried umbilical cord, newborn urine, saliva, or serum collected within 21 days of life or from the newborn dried blood spot.

#### Comparators

Children with cCMV infection will be compared either with non-infected children or, in subgroup analyses, we will compare symptomatic and asymptomatic cCMV populations.

#### Outcomes

The primary outcome will be the effect of cCMV infection on neurodevelopmental outcomes in children assessed using standardized and validated neurodevelopmental instruments. These may include comprehensive developmental assessment scales, such as the Bayley Scales of Infant and Toddler Development [5], as well as developmental screening or neurological assessment tools, including the Ages and Stages Questionnaire (ASQ-3) [6], Parents’ Evaluation of Developmental Status (PEDS) [7], General Movements Assessment (GMA) [8], Hammersmith Infant Neurological Examination (HINE) [9], and Denver Developmental Screening Test [10]. Outcomes related to cognitive, motor, language, behavioral, and neurological development will be considered. Neurodevelopmental assessments performed at any age up to 18 years will be eligible and, where feasible, analyses will be stratified according to developmental stage or age at assessment.

Secondary outcomes will include growth parameters, hospital readmissions, hearing impairment, visual impairment, and behavioral outcomes. Growth outcomes will include anthropometric measures such as weight, length/height, and head circumference assessed according to age-standardized paediatric growth parameters. Hearing and visual impairment will be defined according to clinical and/or instrumental evaluations reported by the included studies. Behavioral outcomes will include behavioral or psychosocial difficulties assessed through standardized questionnaires or neurobehavioural assessment tools where available. Hospital readmissions related to clinical complications during follow-up will also be considered. Secondary outcomes assessed at any age up to 18 years will be eligible, and timing of assessment will be extracted and categorized according to developmental stage whenever feasible. Quantitative synthesis for these endpoints will be performed only if sufficient methodological and clinical homogeneity across studies is identified.

### Study design

Non-randomized, cross-sectional, or longitudinal studies evaluating neurodevelopmental outcomes in cCMV-infected children aged ≤18 years old cross-sectionally and/or longitudinally will be included.

### Information sources and search strategy

The main databases to be searched are *Embase*—*Embase* via *Ovid*, *MEDLINE*, *PsycInfo*, and *Scopus* exploring two concepts that are cCMV and neurodevelopmental outcomes*.* No limitation in time range will be applied to capture all the findings that include neurodevelopmental outcomes in cCMV children.

Search terms will include controlled vocabulary and free-text keywords related to congenital cytomegalovirus infection and neurodevelopmental impairment, including but not limited to “congenital cytomegalovirus infection,” “CMV,” “neurodevelopmental assessment,” “neurodevelopmental outcomes,” “developmental delay,” and “neurological sequelae.” The search strategy will be adapted for each database as appropriate. In addition, the reference lists of all included studies and relevant reviews will be manually screened to identify further eligible studies. An example search strategy for MEDLINE is as follows: (“congenital cytomegalovirus” OR “cCMV” OR “CMV infection”) AND (“neurodevelopment” OR “neurodevelopmental outcomes” OR “developmental delay” OR “neurological sequelae” OR “cognitive impairment” OR “motor development”).

The literature search strategy was developed in collaboration with a Scientific Information Specialist from Maastricht University to enhance methodological rigour and comprehensiveness.

### Data management

Literature search’s results will be shared among all the authors to approve the eligibility of selected studies according to the eligibility criteria. All authors will pay attention to ascertain duplicate publications or multiple reports of the same study. All records retrieved from the database searches will be imported into Rayyan (Rayyan Systems Inc., Cambridge, MA, USA), a web-based platform designed to support systematic review screening and study selection. Duplicate records will be identified and removed prior to screening. Two reviewers will independently screen titles and abstracts for eligibility, followed by full-text assessment of potentially relevant studies. Rayyan’s blinding and conflict-resolution features will be used to facilitate independent screening and identification of disagreements between reviewers. Any discrepancies will be resolved through discussion and consensus or, when necessary, consultation with a third reviewer.

### Selection process

Two reviewers will independently select eligible articles evaluating the titles and abstracts and verifying the acceptability based on the eligibility criteria. Any disagreements will be resolved through discussion and consensus; if consensus cannot be reached, a third reviewer will adjudicate. Results will be compared and discussed among all the authors.

### Data collection process and items

Information will be extracted using a standardized form and reported in a Microsoft Excel (Redmond, WA: Microsoft, 2013) spreadsheet. Prior to formal data extraction, the standardized data extraction form will be pilot tested on a sample of eligible studies to assess its clarity, completeness, and suitability for consistent data collection. Necessary modifications will be made before full data extraction is initiated. The following data will be extracted from the studies: author, year of publication, number of subjects included, cCMV status and diagnostic method used, age at the assessment, neurodevelopmental domains measured, neurodevelopmental tests used, comparison group included yes/no, odds ratios/mean scores/*z*-scores, cCMV predictors of neurodevelopmental outcome, additional outcomes.

Studies with high suspicion of selective reporting bias (SRB) will be processed as follows: authors will be asked to provide any required data not available in published reports.

Two authors will do this process independently, in duplicate. Any disagreements will be resolved through discussion and consensus; if consensus cannot be reached, a third reviewer will adjudicate. The results will be compared and discussed among all the authors.

### Risk of bias in included studies

Risk of bias in non-randomized studies will be independently assessed by two reviewers using the Newcastle-Ottawa Scale (NOS) [11], complemented where appropriate by the ROBINS-I tool [12] to provide a more comprehensive evaluation of methodological quality and potential sources of bias. The NOS evaluates three main domains: selection of study groups, comparability of groups, and ascertainment of exposure and outcomes. ROBINS-I assesses potential bias across multiple domains, including confounding, participant selection, classification of interventions/exposures, missing data, outcome measurement, and selective reporting. Each domain will be judged according to the recommendations of the respective tools, and an overall risk-of-bias judgement will be assigned for each included study. Any disagreements between reviewers will be resolved through discussion and consensus or, if necessary, consultation with a third reviewer.

Publication bias will be evaluated, where feasible, through visual inspection of funnel plots and statistical methods such as Egger’s regression test when a sufficient number of studies are included in the meta-analysis.

### Data synthesis

Dichotomous outcomes will be summarized using log odds ratios (logORs), whereas continuous neurodevelopmental outcomes will be pooled using standardized mean differences (Hedges’ *g*). Corresponding standard errors will be calculated for all effect estimates to allow quantitative synthesis.

Because neurodevelopmental outcomes may be assessed using different validated instruments across studies, continuous outcomes will be harmonized using Hedges’ *g* standardized effect sizes and corresponding standard errors, enabling pooling across different neurodevelopmental scales while accounting for small-sample bias. Whenever available, age-standardized normative scores (e.g., Bayley composite scores, standardized scores, or developmental quotients) will be preferentially extracted rather than raw scores. For instruments in which higher scores indicate worse performance, outcome directions will be reversed prior to calculating Hedges’ *g* to ensure consistency of effect direction across studies.

Meta-analyses will be conducted separately according to neurodevelopmental domain (e.g., cognitive, language, and motor outcomes). In addition, subgroup and sensitivity analyses stratified by assessment instrument and, where feasible, by age at assessment will be performed to explore residual conceptual heterogeneity across instruments.

Values of logOR and Hedges’ *g* will be pooled and analyzed by a Bayesian model-averaged (BMA) meta-analysis [13–15]. We chose BMA and robust Bayesian meta-analysis (RoBMA) because these approaches allow us to quantify evidence both for the presence and for the absence of an effect, while formally incorporating uncertainty regarding key meta-analytic assumptions, including fixed versus random effects and the presence versus absence of publication bias. In contrast to conventional frequentist random-effects meta-analysis, which relies primarily on dichotomous *p *value thresholds, Bayesian approaches provide a continuous measure of evidential strength and can distinguish between “absence of evidence” and “evidence of absence” when findings are imprecise or statistically non-significant. RoBMA further improves robustness by averaging across multiple publication-bias adjustment models and weighting them according to their predictive performance, thereby avoiding all-or-none assumptions regarding publication bias and yielding more stable inferences under heterogeneity and potential selective reporting.

We will perform the analysis in JASP, which utilizes the RoBMA R package [16, 17]. BMA employs Bayes factors (BFs) and Bayesian model-averaging to evaluate the likelihood of the data under the combination of models assuming the presence vs. the absence of the meta-analytic effect and heterogeneity [13, 14, 17]. The BF_10_ is the ratio of the probability of the data under H_1_ over the probability of the data under H_0_. Therefore, the BF_10_ represents how much more likely the observed data are under the hypothesis that an effect exists (H_1_) than under the null hypothesis of no effect (H_0_). For example, BF_10_ = 5 indicates that the data are five times more likely under H_1_ than under H_0_, whereas BF_10_ = 0.2 (equivalently, BF_01_ = 5) indicates that the data are five times more likely under H_0_ than under H_1_. We will report BF_10_ (or BF_01_) alongside pooled effect estimates and 95% credible intervals (CrI). Following the evidence categories suggested by Lee and Wagenmakers [18], BF_10_ values between 1 and 3 will be considered weak/inconclusive evidence, 3–10 moderate evidence, 10–30 strong evidence, 30–100 very strong evidence, and >100 extreme evidence in favor of H_1_; reciprocal values will indicate corresponding levels of evidence in favor of H_0_. The BF_rf_ is the ratio of the probability of the data under the random-effects model over the probability of the data under the fixed-effects model.

Publication bias will be formally assessed and, where supported by the available data, adjusted for using RoBMA [19]. RoBMA is a Bayesian model-averaging framework that simultaneously evaluates evidence regarding (i) the presence or absence of an overall effect, (ii) between-study heterogeneity, and (iii) publication bias by averaging across a prespecified ensemble of models with different assumptions about selective reporting and small-study effects. Rather than relying on a single publication-bias correction method, RoBMA integrates evidence across multiple complementary approaches, including selection models and small-study-effect adjustments, and weights them according to their posterior predictive performance. This approach yields a model-averaged bias-adjusted pooled estimate with corresponding 95% credible intervals, as well as an inclusion Bayes factor for publication bias (BF_bias_) quantifying the strength of evidence supporting the presence versus absence of publication bias [19].

Bayesian model-averaged meta-regression (BMA-reg) will be performed to explore potential sources of between-study heterogeneity and to investigate whether specific clinical or methodological characteristics moderate neurodevelopmental outcomes in children with cCMV infection. Potential moderators will include symptomatic versus asymptomatic status at birth, age at neurodevelopmental assessment, neurodevelopmental domain, assessment instrument, and study design, provided that sufficient data are available. BMA-reg combines models with different assumptions regarding the presence or absence of an effect, heterogeneity, and moderation, thereby allowing evaluation of the evidence for or against each component while accounting for uncertainty in the remaining components [20]. Evidence for moderation will be quantified using BFs for moderation (BF_mod_).

The categories of strength of the evidence in favor of the random effects (BF_rf_ > 1) or the fixed effect (BF_rf_ < 1), presence vs. absence of publication bias (BF_bias_ > 1 vs. BF_bias_ < 1), or presence vs. absence of moderation by GA (BF_mod_ > 1 vs. BF_mod_ < 1) were similar to those described above for the BF_10_.

For all the analyses, we will use the neonatal-specific empirical prior distributions based on the Cochrane Database of Systematics Reviews [13, 17, 19]. Binary outcomes: logOR ~ Student-*t* (*μ* = 0, *σ* = 0.29, *ν* = 3), tau(logRR) ~ Inverse-Gamma (*k* = 1.80, *θ* = 0.42); continuous outcomes: Cohen’s *d* ~ Student-*t* (*μ* = 0, *σ* = 0.42, *ν* = 5), tau(Cohen’s *d*) ~ Inverse-Gamma (*k* = 1.68, *θ* = 0.38) [13, 20, 21]. These priors are implemented in JASP and are empirically derived and calibrated using large sets of Cochrane meta-analyses, with out-of-sample evaluation of predictive performance, as described by Bartoš et al. [13, 17, 19]. As a prior sensitivity analysis [19], we will rerun the BMA meta-analysis using the corresponding general (field-wide) empirical priors from the same Bartoš et al. framework. This comparison directly evaluates whether posterior estimates and Bayes factors are robust to the choice between subdiscipline-specific versus general Cochrane-calibrated priors, while maintaining a consistent prior-construction methodology (i.e., both priors are empirical, Cochrane-based, and implemented within the same model-averaging architecture).

For the meta-regression, we will use the prior distributions for the effect size and heterogeneity parameter with the prior distribution on the standardized meta-regression coefficient scaled by 1/2 (i.e., the expected difference in each moderator level corresponding to 1/2 of the mean effect size) [19]. To test the robustness of this analysis, another meta-regression will be conducted with the prior distribution on the standardized meta-regression coefficient scaled by 1/4.

Posterior distributions will be approximated using Markov chain Monte Carlo (MCMC) sampling with 3 chains, 2000 adaptation iterations, 2000 burn-in iterations, 5000 posterior samples, and thinning set to 1. Convergence will be assessed using the default JASP/RoBMA diagnostics, including the potential scale reduction factor (R-hat ≤ 1.05) and effective sample size (ESS ≥ 500) [17]. The corresponding computational code is provided as supplementary material to ensure reproducibility.

### Confidence in cumulative evidence

The strength of the body of evidence will be assessed as follows:

- For the interpretation of the study contribution to the findings, references will be made to the prior assessment of methodological quality of the included studies.
- Consistency and inconsistency across findings will be assessed and any incoherent or contradictory evidence will be highlighted and discussed.
- The certainty and overall quality of evidence for quantitative outcomes included in the meta-analysis will be assessed using the GRADE (Grading of Recommendations Assessment, Development and Evaluation) framework. Evidence quality will be evaluated across the domains of risk of bias, inconsistency, indirectness, imprecision, and publication bias, and categorized as high, moderate, low, or very low certainty. The GRADE assessment will be performed independently by two reviewers, with disagreements resolved through discussion and consensus. The CERQual approach will be considered only for qualitative findings, if qualitative evidence synthesis is performed.

## Discussion

To the best of our knowledge, this systematic review will be the first to sum up the current evidence on the neurodevelopmental outcomes in children with cCMV. The protocol illustrates the methodology that will serve to rigorously extract, screen, and compute the results of the selected studies. On one hand, the systematic review will quantitatively document the predictive role of cCMV on the neurodevelopment of infected and non-infected children to define strengths and weaknesses of each group. On the other hand, the meta-analytic approach will define the robustness of the findings from the selected studies. Overall, our findings will contribute to improve guidelines of care for these groups of children and to implement counseling and early interventions tailored on specific neurodevelopmental domains. We anticipate the potential challenges in carrying out the meta-analytic computation due to the low number of eligible studies and their heterogeneity.

## Supplementary Information

Additional file 1: PRISMA-P 2015.

Additional file 2: Computational code.

## Data Availability

The availability of the dataset that will be generated during and analysed during this systematic review shall be reported accordingly after the systematic review is done. It is sufficient to mention that the data shall be found in the databases mentioned above.

## Abbreviations

ASQ-3: Ages and Stages Questionnaire
BF: Bayes factor
BMA-reg: Bayesian model-averaged meta-regression
cCMV: Congenital cytomegalovirus infection
GMA: General Movements Assessment
HINE: Hammersmith Infant Neurological Examination
PEDS: Parents’ Evaluation of Developmental Status
PRISMA-P: Preferred Reporting Items for Systematic Review and Meta-Analysis Protocols
RoBMA: Robust Bayesian meta-analysis
SRB: Selective reporting bias
