# Supplementary material for: Neurodevelopmental outcome in children with congenital cytomegalovirus infection: a systematic review and meta-analysis protocol
Source: Syst Rev. 2026 Aug 8;15:286. doi: 10.1186/s13643-026-03288-6 (PMC13737908; doi:10.1186/s13643-026-03288-6)
Supplement: Supplementary file 2 [file 13643_2026_3288_MOESM2_ESM.docx]

**Supplementary Code 1. Bayesian model-averaged meta-analysis for continuous outcomes**

fit <- NoBMA(
d = dataset[['computed effect size']],
se = dataset[['computed standard error']],
prior_scale = 'cohens_d',
transformation = 'fishers_z',
priors_effect = list(
prior(distribution = 't', parameters = list(location = 0, scale = 0.42, df = 5), truncation = list(lower = -Inf, upper = Inf), prior_weights = 1)
),
priors_heterogeneity = list(
prior(distribution = 'invgamma', parameters = list(shape = 1.68, scale = 0.38), truncation = list(lower = 0, upper = Inf), prior_weights = 1)
),
priors_effect_null = list(
prior(distribution = 'point', parameters = list(location = 0), prior_weights = 1)
),
priors_heterogeneity_null = list(
prior(distribution = 'point', parameters = list(location = 0), prior_weights = 1)
),
chains = 3,
adapt = 2000,
burnin = 2000,
sample = 5000,
thin = 1,
autofit = TRUE,
autofit_control = list(max_Rhat = 1.05, min_ESS = 500, max_error = NULL, max_SD_error = NULL, max_time = NULL, sample_extend = 5000, restarts = 10, max_extend = 10),
algorithm = 'ss'
)

**Supplementary Code 2. Bayesian model-averaged meta-analysis for dichotomous outcomes using the log odds ratio scale**

fit <- NoBMA(
logOR = dataset[['computed effect size']],
se = dataset[['computed standard error']],
prior_scale = 'logOR',
transformation = 'fishers_z',
priors_effect = list(
prior(distribution = 't', parameters = list(location = 0, scale = 0.29, df = 3), truncation = list(lower = -Inf, upper = Inf), prior_weights = 1)
),
priors_heterogeneity = list(
prior(distribution = 'invgamma', parameters = list(shape = 1.8, scale = 0.42), truncation = list(lower = 0, upper = Inf), prior_weights = 1)
),
priors_effect_null = list(
prior(distribution = 'point', parameters = list(location = 0), prior_weights = 1)
),
priors_heterogeneity_null = list(
prior(distribution = 'point', parameters = list(location = 0), prior_weights = 1)
),
chains = 3,
adapt = 2000,
burnin = 2000,
sample = 5000,
thin = 1,
autofit = TRUE,
autofit_control = list(max_Rhat = 1.05, min_ESS = 500, max_error = NULL, max_SD_error = NULL, max_time = NULL, sample_extend = 5000, restarts = 10, max_extend = 10),
algorithm = 'ss'
)
